# Modification by *ALAD* of the Association between Blood Lead and Blood Pressure in the U.S. Population: Results from the Third National Health and Nutrition Examination Survey

**DOI:** 10.1289/ehp.0900866

**Published:** 2009-10-07

**Authors:** Franco Scinicariello, Ajay Yesupriya, Man-huei Chang, Bruce A. Fowler

**Affiliations:** 1 Division of Toxicology and Environmental Medicine, Agency for Toxic Substances and Disease Registry, Atlanta, Georgia, USA; 2 Office of Public Health Genomics, Coordinating Center for Health Promotion, Centers for Disease Control and Prevention, Atlanta, Georgia, USA

**Keywords:** ALAD polymorphism, blood pressure, hypertension, lead, NHANES

## Abstract

**Background:**

Environmental lead exposure has been found to be associated with an increased risk of hypertension. Individuals vary greatly in susceptibility to lead toxicity, and genetic susceptibility has often been cited as the probable cause for such variation.

**Objective:**

The main objective is to determine the role of the aminolevulinic acid dehydratase (*ALAD*) gene, which encodes the main carrier protein of lead in blood, in the association between lead exposure and blood pressure (BP) and hypertension in the U.S. population.

**Methods:**

We analyzed data from individuals ≥ 17 years of age who participated in the Third National Health and Nutrition Examination Survey for whom DNA was available (*n* = 6,016). Multivariable logistic and linear regressions stratified by race/ethnicity were used to examine whether hypertension and BP were associated with *ALAD* and blood lead levels (BLL).

**Results:**

BLL was associated with systolic BP in non-Hispanic whites and with hypertension and systolic and diastolic BP in non-Hispanic blacks. BLL was not associated with BP outcomes in Mexican Americans. Non-Hispanic white *ALAD2* carriers in the highest BLL quartile (3.8–52.9 μg/dL) had a significantly higher adjusted prevalence odds ratio for hypertension compared with *ALAD1* homozygous individuals. We also found a significant interaction between lead concentration and the *ALAD2* allele in non-Hispanic whites and non-Hispanic blacks in relation to systolic BP.

**Conclusions:**

BLL may be an important risk factor for hypertension and increased systolic and diastolic BP. These associations may be modified by *ALAD* genotype.

Occupational studies have repeatedly demonstrated that blood lead levels (BLL) > 40 μg/dL are associated with increased risk of elevated blood pressure (BP) (reviewed by Navas-Acien et al. 2007). A recent meta-analysis reported that a 2-fold increase in BLL was associated with a 1.0-mmHg and a 0.6-mmHg increase in systolic and diastolic BP, respectively (Nawrot et al. 2002). Previous studies have demonstrated associations between low-level lead exposure and increased BP (Den Hond et al. 2002; Nash et al. 2003; Navas-Acien et al. 2004; Nordberg et al. 2000; Pirkle et al. 1985; Vupputuri et al. 2003), but the results have been uneven, suggesting that other biological factors (e.g., genetic and race/ethnicity) may also be operating.

The δ-aminolevulinic acid dehydratase (ALAD) enzyme catalyzes the second step in heme biosynthesis and is known to be the major carrier protein for lead in blood (Bergdahl et al. 1997). ALAD, which in humans is encoded by a single gene localized to the chromosome 9q34 region, is a polymorphic enzyme with two co-dominantly expressed alleles, *ALAD1* and *ALAD2* (dbSNP ID rs1800435) (Battistuzzi et al. 1981). The difference between these two alleles lies in a single G→C transversion mutation of nucleotide 177 in *ALAD2*; the allozyme resulting from the *ALAD2* allele contains the substitution of a neutral asparagine for a positively charged lysine at residue 59 (Wetmur et al. 1991). Three differently charged allozymes, ALAD1‐1, 1-2, and 2-2, result from the expression of the *ALAD1* and *ALAD2* alleles, which have different affinities for lead (Bergdahl et al. 1997). The frequencies of the *ALAD1* and *ALAD2* alleles in several white populations have been estimated to be 0.9 and 0.1, respectively, whereas Asian and African populations have lower *ALAD2* allele frequencies (Kelada et al. 2001). It is well known that individuals vary greatly in susceptibility to lead toxicity, and genetic susceptibility has often been cited as the probable cause for such variation (Kelada et al. 2001). In a review of occupational studies in which lead exposure was relatively high, the rs1800435 polymorphism in the *ALAD* gene was positively associated with BLL; however, no association has been found between *ALAD* and BLL among environmentally exposed adults with BLL < 10 μg/dL (Scinicariello et al. 2007).

Few studies have addressed the relationship between *ALAD* genotype status and BLL and BP, and the results have been inconclusive. A study conducted among Korean lead smelter workers (*n* = 798; mean BLL = 32.0 μg/dL) showed that *ALAD2* carriers had a statistically significantly increased systolic BP at occupational lead exposure levels compared with *ALAD1* homozygous carriers (Lee et al. 2001). A previous study conducted among 691 members of a construction trade union (mean BLL = 7.78 μg/dL) found that systolic BP and diastolic BP were increased in *ALAD2* carriers compared with members homozygous for the *ALAD1* allele. This difference, however, was not statistically significant (Smith et al. 1995).

Currently, the Centers for Disease Control and Prevention (CDC) designates 10 μg/dL as a BLL of concern and has formulated guidelines for environmental and educational intervention in children at this level (CDC 2002). However, no corresponding CDC guidance exists for BLL measured in adults.

The objective of our study is to determine, in a large, nationally representative sample [Third National Health and Nutrition Examination Survey (NHANES III), 1988–1994], whether the relationship between BP and lead exposure differs by *ALAD* genotype status.

## Materials and Methods

NHANES III is a survey representative of the civilian, noninstitutionalized U.S. population and was conducted in 1988–1994 (National Center for Health Statistics 1994). The sample was selected through a complex, multistage probability design and included oversampling of non-Hispanic blacks, Mexican Americans, children, and the elderly (National Center for Health Statistics 1994). Sample weights account of differential probability of selection and nonresponse and are poststratified to census population estimates. Individuals participated in an interview conducted at home and also received an extensive physical examination performed at a mobile examination center (MEC), which included blood and urine collection. The total number of participants from the second phase of NHANES III (1991–1994) was 16,530. The NHANES III DNA bank contains specimens from 7,159 participants ≥ 12 years of age who were examined during the second phase of NHANES III (1991–1994) (Chang et al. 2009). The sample weights were derived from the NHANES III phase 2 MEC-examined sample weights, and they were recalculated using previously described methods (Lohr 1999) for the 7,159 participants for whom DNA was available to avoid nonresponse bias for the NHANES III genetic data.

The present study included participants in the NHANES III DNA bank ≥ 17 years of age and self-reported as non-Hispanic white, non-Hispanic black, or Mexican American (*n* = 6,016). Further details regarding the NHANES III DNA bank have been previously described (Chang et al. 2009).

### Definitions and measurements of variables

#### Outcome variables

Hypertension status and two BP measures (systolic and diastolic BP) were the outcome measures. Hypertension was defined as self-reported current use of an antihypertensive medication, systolic BP of ≥ 140 mmHg, or diastolic BP of ≥ 90 mmHg. Systolic and diastolic BP levels were examined for persons not currently taking antihypertensive medication. Hypertension was treated as a dichotomous variable, and the two BP measures were treated as continuous variables.

BP for individual participants was calculated as the average of all available measurements (maximum = 6) from the home interview and MEC.

BP measurements were performed in the MEC by the physician on children (5–19 years of age) and adults (≥ 20 years of age); the interviewers took measurements on adults only (≥ 17 years of age) in the household. More details can be found at the reference manual of BP quality control program (CDC 1991).

#### Other variables

BLL was determined using graphite furnace atomic absorption spectrophotometry. Total serum calcium was measured by ion-selective electrodes. Serum creatinine was measured by the modified kinetic Jaffe reaction using a Hitachi 737 analyzer (Boehringer Mannheim Corp., Indianapolis, IN). Glycosylated hemoglobin (HbA1C) was measured using ion exchange chromatography. Details of the laboratory protocols for each of these measures can be found on the CDC National Center for Health Statistics Web site (Gunter et al. 1996).

Phenotypic covariates analyzed in the regression analysis included the continuous variables of body mass index (BMI, calculated as weight in kilograms divided by the square of height in meters) and serum creatinine levels and the categorical variables of age (17–39, 40–59, and ≥ 60 years), sex, education (< high school, completed high school, some college), cigarette smoking history (current, former, or never), and weekly alcohol intake (none, < 4, and ≥ 4 drinks per week).

### ALAD genotyping methods

The *ALAD* polymorphism was genotyped at the Core Genotyping Facility, National Cancer Institute, National Institutes of Health (Bethesda, MD), using TaqMan assays (Applied Biosystems, Foster City, CA). Detailed information on genotyping methods and quality control methods have been previously described (Chang et al. 2009). Briefly, the quality of the genetic data was assured through the use of water controls, DNA samples with known genotypes, blinded replicates of approximately 6% of samples, and tests for deviations from Hardy–Weinberg proportions.

### Statistical analysis

We used multivariable logistic and linear regression to examine the relationships among hypertension (and BP measures), BLL, and the *ALAD* polymorphism in non-Hispanic white, non-Hispanic black, and Mexican-American race/ethnicity categories. Participants were categorized into BLL quartiles based on the weighted population distribution. The *ALAD* polymorphism was assessed assuming a dominant model (*ALAD1*/*ALAD1* vs. *ALAD1*/*ALAD2* and *ALAD2*/*ALAD2*) where the homozygous major allele (*ALAD1*) was the reference group and the heterozygous plus homozygous for the minor allele (*ALAD2*) was the evaluated group.

Models were analyzed separately for the three outcomes of interest as indicated above. Effect modification by *ALAD* in the relationship between blood levels and hypertension outcome was examined through models stratified by BLL quartiles: BLL = 0.7–1.4, 1.5–2.3. 2.4–3.7. 3.8–52.9 μg/dL. Also, analyses were run excluding from the fourth quartile individuals with BLL > 10.0 μg/dL (BLL range, 3.8–10.0 μg/dL).

We used the multivariable linear regression to examine the relationships among BP measures (systolic and diastolic BP), *ALAD* genotype status, log-transformed BLL, and the interaction term between log-transformed BLL and *ALAD* genotype status. The BLLs were log-transformed (natural logarithms) because the lead levels in blood were right skewed. The change in BP associated with a doubling of the BLL was calculated by multiplying the regression coefficient by 0.69 (Nawrot et al. 2002).

We conducted multivariable regression models adjusting for the potential risk factors age, sex, education, smoking status, alcohol intake, BMI, serum creatinine levels (as a marker of kidney function), serum calcium, glycosylated hemoglobin (a time-integrated marker of average glycemia during the previous 3 months), and hematocrit. These risk factors have previously shown to be associated with BP and hypertension (Burt et al. 1995) and with lead and BP (Nash et al. 2003; Vupputuri et al. 2003), including hematocrit (Hense et al. 1993) and alcohol intake (Hense et al. 1993, 1994).

Statistical analyses were performed using SAS version 9.1 (SAS Institute Inc., Cary, NC) and SAS-callable SUDAAN version 9.01 (Research Triangle Institute, Research Triangle Park, NC) to account for the NHANES III complex sample design. *p*-Values from Satterthwaite statistics were presented at the significance level of 0.05.

## Results

Table 1 illustrates the characteristics of participants (*n* = 6,016) from the NHANES III DNA bank weighted to be representative of the U.S. population. The mean age of the population was 44 years (data not shown). Approximately 52% of individuals were female. Non-Hispanic whites accounted for 81.6% of the total population. Approximately 43%, 47%, and 49% of the people reported that they had attended some college, never used alcohol, and never smoked, respectively. The mean (± SE) for systolic BP was 119.0 ± 0.39 mmHg and for diastolic BP was 73.5 ± 0.27 mmHg. Hypertension was observed in 22.7% of the population. For those not currently treated for hypertension, 9.2% and 5.5%, had systolic (≥ 140 mmHg) and diastolic (≥ 90 mmHg) hypertension, respectively. We estimated the mean (± SE) BLL to be 2.98 ± 0.09 μg/dL. Table 1 also summarizes the characteristic of the study participants by race/ethnicity.

Table 2 summarizes the characteristics of the study participants by BLL quartile. Individuals in the highest BLL quartiles tended to be older, male, current smokers, regular drinkers, less educated, and more likely to have hypertension and to have increased serum creatinine, hematocrit, and systolic and diastolic levels of BP.

Table 3 presents the *ALAD* genotypes and mean BLL by *ALAD* genotype and by BLL quartile for each of the three major race/ethnic groups in the United States. *ALAD2* carriers comprised 13.6% of the total population, 15.6% (370 of 2,017) of non-Hispanic whites, 2.6% (49 of 1,621) of non-Hispanic blacks, and 8.8% (137 of 1,609) of Mexican Americans. Among participants with the *ALAD1-1* genotype, we observed a slightly higher BLL for all race/ethnic subgroups compared with the participants with *ALAD1‐2* or *ALAD2‐2* genotypes. However, this association was significant only in non-Hispanic blacks in the third BLL quartile.

### Hypertension

Table 4 shows adjusted prevalence odds ratio (POR) for hypertension for each of the three major race/ethnic subgroups in the U.S. population. We observed no difference between BLL quartile and hypertension in non-Hispanic whites and Mexican Americans. However, in the non-Hispanic black population, individuals in the second, third, and fourth BLL quartiles had a significant adjusted POR (1.83, 2.38, and 2.93, respectively) for hypertension compared with the lowest quartile. We found no significant associations of *ALAD* and hypertension in any of the race/ethnic groups.

Table 5 presents the adjusted PORs for hypertension comparing *ALAD2* carriers and *ALAD1* homozygous individuals stratified by BLL quartile and by race/ethnicity. In the non-Hispanic white population, *ALAD2* carriers in the highest BLL quartile (3.8–52.9 μg/L) had a significantly higher POR [2.00; 95% confidence interval (CI), 1.12–3.55] of hypertension than did the subjects who were *ALAD1* homozygous. Moreover, when we excluded from the fourth quartile those with BLL > 10.0 μg/dL, we still observed a significant increase in risk of hypertension (POR = 1.86; 95% CI%, 1.00–3.49) (data not shown).

### Systolic and diastolic BP

We used multivariate linear regression models to assess associations with systolic and diastolic BP in each race/ethnic group for individuals who were not currently taking medications for high BP (Table 6). We found a significant interaction between BLL and *ALAD* in relation to systolic BP in two race/ethnic subgroups (*p* = 0.04 for non-Hispanic whites, and *p* = 0.02 for non-Hispanic blacks). In multivariate regression analyses for diastolic BP, the interaction between *ALAD* and BLL was not significant in any of the three race/ethnic groups (Table 6).

## Discussion

Our results for the association of blood lead with BP and with hypertension in the race/ethnic groups in the U.S. population is consistent with previous NHANES III studies (Den Hond et al. 2002; Nash et al. 2003; Vupputuri et al. 2003). Estimates from the coefficient regressions for blood lead (Table 6) predicted that a 2-fold increase of BLL was correlated with increases of 1.76 mmHg (95% CI, 1.06–2.47) and 0.72 mmHg (95% CI, 0.19–1.26) in systolic BP for non-Hispanic blacks and whites, respectively. Although we did not observe an association with BLL and hypertension in non-Hispanic whites and Mexican Americans, we did find an increased prevalence of hypertension in non-Hispanic blacks in the second, third, and fourth lead quartile compared with those in the lowest BLL quartile. A previous evaluation of lead levels and hypertension in the NHANES III population by Vupputuri et al. (2003) also showed that black and white women with BLL ≥ 5 μg/dL had statistically significantly higher odds ratio for having hypertension compared with those with BLL < 5 μg/dL.

We found that within the highest quartile of lead for the non-Hispanic white population, the prevalence of hypertension was significantly higher among *ALAD2* carriers compared with *ALAD1* homozygote individuals. In addition, estimates from regression coefficients of the interaction terms shown in Table 6 indicate that, for a doubling of BLL, systolic BP increased by an estimated 2.46 mmHg for *ALAD1‐2*/*2‐2* individuals and 0.72 mmHg for *ALAD1* homozygous individuals for the non-Hispanic white population. In contrast, for non-Hispanic black individuals, for a doubling of BLL, systolic BP decreased by an estimated 4.04 mmHg for *ALAD1-2*/*2-2* individuals and increased by 1.76 mmHg for *ALAD1* homozygous individuals. This finding may not be reliable for non-Hispanic blacks because there were substantially fewer *ALAD2* carriers in this population in our study (*n* < 50).

Of the two previous studies on *ALAD*, BLL, and BP, one reported an increase in systolic BP among Korean *ALAD2* carriers who were occupationally exposed to lead (Lee et al. 2001), whereas the other reported that ALAD was not associated with systolic BP (Smith et al. 1995).

The mechanisms of lead-induced hypertension are not well characterized. One hypothesis is that lead induces hypertension through direct effects on the kidney (Muntner et al. 2003). Another hypothesis is that the accumulation of lead in the walls of arteries results in arterial stiffness, which induces hypertension. Lead has been reported to both accumulate in the human aorta (Poklis 1975; Schroeder and Tipton 1968) and contribute to the increase in pulse pressure that occurs with aging (Perlstein et al 2007). Elevated aortic stiffness is also known to induce high systolic BP and increase pulse pressure (O’Rourke and Mancia 1999). Finally, it is also possible that lead may alter BP by interference with vascular signaling pathways. Endothelial nitric oxide (NO) regulates vascular function, and the disruption of the NO activity is important in the development of hypertension (Chowdhary and Townend 2001). Lead exposure has been reported to significantly inhibit endothelial NO production (Barbosa et al. 2006), as well as to cause NO inactivation by increasing oxidative stress, thereby decreasing NO availability (Vaziri and Ding 2001; Vaziri et al. 1999).

There was no association between *ALAD2* carriers and mean BLL in any of the race/ethnicities. However, in general, *ALAD2* carriers had a lower mean BLL than did *ALAD1* homozygous subjects, although these differences were not statistically significant. When stratified by BLL quartile, only non-Hispanic white *ALAD2* carriers had a marginally higher mean BLL than did *ALAD1* homozygous subjects in the fourth BLL quartile. These data are in agreement with the view that *ALAD2* allele would significantly affect BLL not at low exposure levels but only at higher levels, when other lead-binding sites have been saturated (Schwartz et al. 1995; Scinicariello et al. 2007). Montenegro et al. (2006) reported that, although *ALAD2* carriers had no significantly lower mean BLL than did *ALAD1* homozygous individuals, *ALAD2* carriers had a significantly higher plasma lead level compared with homozygous *ALAD1* subjects. Therefore, it may be possible that non-Hispanic white *ALAD2* carriers have higher levels of plasma lead compared with *ALAD1* homozygote individuals. Consequently, the higher plasma lead, interacting with other molecular BP regulatory systems, may be responsible for the observed increases in systolic BP in *ALAD2* carriers.

The present study has several limitations. The exclusion from our study of persons who reported taking medication for hypertension may have diluted the strength and magnitude of associations in our analysis for systolic and diastolic BP. Second, although we controlled for many of the known factors associated with BP and hypertension, other variables such as serum selenium (Telisman et al. 2001), serum zinc (Schwartz 1991), and blood cadmium (Navas-Acien et al. 2004) might have influenced our findings. Blood cadmium was not measured by NHANES III. However, urinary cadmium, a measured variable in NHANES III, was not a significant variable for hypertension or for systolic or diastolic BP (data not shown). Urinary cadmium reflects cadmium concentration in the renal cortex and is a biomarker of both ongoing and long-term cadmium exposure, whereas blood cadmium is a biomarker of ongoing exposure (Agency for Toxic Substances and Disease Registry 1999). Therefore, it seems unlikely that including blood cadmium in our models would have changed our finding. Our results are based on BLL; because approximately 95% of the total body burden of lead is present in the skeleton, a preferred measure of chronic body burden would be bone lead (Hu et al. 1996). However, the measurement of bone lead in a large sample size, such as NHANES, is not feasible, and blood lead is known to be associated with bone lead (Gwiazda et al. 2005; Todd et al. 2001).

Currently, the CDC designates 10 μg/dL as a BLL of concern and has formulated guidelines for environmental and educational intervention in children at this level (CDC 2002). No corresponding CDC guidance exists for BLL measured in adults. However, in the past few decades the presence of lead in the environment has steadily declined. In the adult U.S. population, mean BLL measured in NHANES surveys conducted in 1976–1980, 1988–1991, and 1999–2002 decreased from 13.1 μg/dL to 3.0 μg/dL and to 01.64 μg/dL, respectively (Muntner et al. 2005; Pirkle et al 1994). This positive and welcome decline has steadily continued with a geometric mean BLL of 1.41 μg/dL in the U.S. adult population ≥ 20 years of age as measured in the NHANES survey conducted in 2005–2006 (Scinicariello F, unpublished data).

## Conclusions

We examined the modification by ALAD in the association of BLL and BP and hypertension in the U.S. population. We found that within the highest quartile of lead for the non-Hispanic white population, the prevalence of hypertension was significantly higher among ALAD2 carriers compared with *ALAD1* homozygotes. In addition, we found that *ALAD2* carriers in non-Hispanic whites may experience a more pronounced effect of lead on systolic BP than do homozygous *ALAD1* carriers. These results underscore the importance of reducing environmental sources of lead exposure in the U.S. population, and this should remain a major public health priority based on consistent evidence of increased health risks (Kosnett et al. 2007; Schwartz and Hu 2007). Given the high frequency of *ALAD2* carriers (15.6%) in the non-Hispanic white population, and the cross-sectional nature of the present study, prospective studies are needed to confirm and elucidate the role of *ALAD* polymorphism and these associations.

## Figures and Tables

**Table 1:** Weighted characteristics of the participants from the NHANES III DNA bank stratified by race/ethnicity.

Characteristic	All participants	Non-Hispanic whites	Non-Hispanic blacks	Mexican Americans
No.	6,016	2,387	1,670	1,746
BLL (μg/dL)	2.99 ± 0.09	2.87 ± 0.09	3.59 ± 0.20	3.33 ± 0.11
*ALAD*1-2/2-2 (%)	13.6	15.6	2.6	8.8
BP (mmHg)				
Systolic BP	119.0 ± 0.39	119 ± 0.48	120.2 ± 0.43	116.9 ± 0.64
Diastolic BP	73.5 ± 0.27	73.5 ± 0.27	74.4 ± 0.47	71.9 ± 0.53
Systolic BP = 140 mmHg (%)^a^	9.2	9.3	10.4	6.3
Diastolic BP = 90 mmHg (%)^a^	5.4	5.1	8.8	4.1
Hypertension (% yes)^b^	22.7	22.6	28.4	13.8
Race/ethnicity (%)				
Non-Hispanic white	81.6			
Non-Hispanic black	12.4			
Mexican American	6.0			
Age [years (%)]				
17–39	47.0	44.4	55.8	65.4
40–59	30.9	31.7	28.3	25.1
≥ 60	22.1	23.9	15.9	9.5
Female (%)	52.1	51.9	55.2	48.5
BMI (kg/m^2^)	26.7 ± 0.16	26.5 ± 0.18	28.0 ± 0.24	27.6 ± 0.10
Smoking history (%)				
Current smoker	25.8	25.6	29.9	20.7
Former smoker	25.3	27.3	14.0	20.3
Never smoker	48.9	47.1	56.1	59.1
Alcohol use (%)				
None	47.4	45.9	55.6	50.8
< 4 drinks/week	27.8	28.8	21.7	25.9
≥ 4 drinks/week	24.8	25.3	22.8	23.4
Education (%)				
< High school	22.7	18.6	33.3	56.9
Completed high school	34.6	34.7	38.4	26.2
Some college	42.7	46.7	28.3	16.9
Serum creatinine (mg/dL)	0.86 ± 0.01	0.86 ± 0.01	0.92 ± 0.01	0.77 ± 0.01
Serum calcium (mg/dL)	9.19 ± 0.03	9.18 ± 0.03	9.22 ± 0.03	8.19 ± 0.04
Hematocrit (%)	42.1 ± 0.12	42.4 ± 0.12	40.6 ± 0.16	42.5 ± 0.15
Log glycosylated hemoglobin (%)	1.68 ± 0.01	1.67 ± 0.01	1.71 ± 0.01	1.69 ± 0.01

Values are percent or mean ± SE.

a Subject with systolic BP of ≥ 140 mmHg or diastolic BP of ≥ 90 mmHg not currently treated for hypertension.

b Hypertension defined as a self-report of currently taking antihypertensive medication, systolic BP of ≥ 140 mmHg, or diastolic BP of ≥ 90 mmHg.

**Table 2:** Characteristics by BLL quartile of adults ≥ 17 years of age participating in the NHANES III DNA bank.

Characteristic	Quartile 1 (0.7–1.4 μg/dL)	Quartile 2 (1.5–2.3 μg/dL)	Quartile 3 (2.4–3.7 μg/dL)	Quartile 4 (3.8–52.9 μg/dL)	*p*-Value for trend
Median BLL (μg/dL)	0.70	1.90	3.00	5.00	
*ALAD2* carriers, *1-2*/*2-2* (%)	15.1	14.2	11.6	13.5	
BP (mmHg)					
Systolic BP	113.5 ± 0.41	116.9 ± 0.46	121.1 ± 0.81	124.6 ± 0.58	< 0.001
Diastolic BP	71.6 ± 0.45	73.0 ± 0.42	74.3 ± 0.44	75.3 ± 0.50	< 0.001
Systolic BP = 140 mmHg (%)^a^	3.0	7.2	11.9	15.1	< 0.001
Diastolic BP = 90 mmHg (%)^a^	3.2	4.6	6.2	8.0	< 0.001
Hypertension, yes (%)^b^	10.9	18.4	27.4	33.1	< 0.001
Race (%)					
Non-Hispanic white	83.7	83.5	81.8	77.6	
Non-Hispanic black	10.8	11.4	12.1	15.4	< 0.01
Mexican American	5.6	5.1	6.1	7.0	
Age [years (%)]					
17–39	71.3	50.6	39.7	28.9	
40–59	21.6	32.6	32.8	35.5	< 0.001
≥ 60	7.1	16.9	27.5	35.6	
Sex (%)					
Male	26.6	40.2	53.8	69.1	< 0.001
Female	73.4	59.8	46.2	30.9	
BMI (kg/m^2^)	26.66 ± 0.47	26.70 ± 0.21	26.70 ± 0.21	26.63 ± 0.22	0.9
Smoking history (%)					
Current	14.2	21.7	29.8	36.6	
Former	19.16	25.4	23.9	32.1	< 0.001
Never	66.7	52.9	46.3	31.3	
Alcohol use (%)					
None	52.5	50.8	46.0	40.6	
< 4 drinks/week	31.6	28.4	27.2	24.3	< 0.001
≥ 4 drinks/week	15.9	20.9	26.8	35.2	
Education (%)					
< High school	16.6	15.6	25.7	32.2	
Completed high school	36.7	33.1	34.2	34.7	< 0.001
Some college	46.7	51.2	40.2	33.1	
Serum creatinine (mg/dL)	0.78 ± 0.01	0.83 ± 0.01	0.88 ± 0.01	0.94 ± 0.01	< 0.001
Serum calcium (mg/dL)	9.15 ± 0.03	9.17 ± 0.03	9.22 ± 0.03	9.21 ± 0.04	0.02
Log glycosylated hemoglobin (%)	1.64 ± 0.01	1.67 ± 0.01	1.70 ± 0.01	1.70 ± 0.01	< 0.001
Hematocrit (%)	40.72 ± 0.24	41.72 ± 0.14	42.83 ± 0.20	43.19 ± 0.16	< 0.001

Values are percent or mean ± SE.

a Subject with systolic BP of ≥ 140 mmHg or diastolic BP of ≥ 90 mmHg not currently treated for hypertension.

b Hypertension defined as a self-report of currently taking antihypertensive medication, systolic BP of ≥ 140 mmHg, or diastolic BP of ≥ 90 mmHg.

**Table 3:** Prevalence of *ALAD* genotypes and BLL (mean ± SE) by race/ethnicity and by BLL quartile.

			BLL quartile			
Genotype	Percent	BLL	Quartile 1 (0.7–1.4 μg/dL)	Quartile 2 (1.5–2.3 μg/dL)	Quartile 3 (2.4–3.7 μg/dL)	Quartile 4 (3.8–52.9 μg/dL)
All participants						
*ALAD1-1*	86.4	3.01 ± 0.09	0.94 ± 0.02	1.88 ± 0.01	3.00 ± 0.04	5.92 ± 0.12
*ALAD1-2*/*2-2*	13.6	2.84 ± 0.14	0.92 ± 0.03	1.84 ± 0.03	2.90 ± 0.04	5.72 ± 0.20
Non-Hispanic whites						
*ALAD1-1*	84.4	2.88 ± 0.09	0.94 ± 0.02	1.88 ± 0.02	3.00 ± 0.02	5.66 ± 0.14
*ALAD1-2*/*2-2*	15.6	2.81 ± 0.14	0.93 ± 0.03	1.83 ± 0.03	2.90 ± 0.04	5.69 ± 0.22
Non-Hispanic blacks						
*ALAD1-1*	97.4	3.58 ± 0.20	0.97 ± 0.02	1.88 ± 0.01	3.02 ± 0.02*	6.89 ± 0.23
*ALAD1-2*/*2-2*	2.6	3.43 ± 0.46	0.87 ± 0.09	1.85 ± 0.08	2.70 ± 0.06	5.86 ± 0.56
Mexican Americans						
*ALAD1-1*	91.2	3.36 ± 0.12	0.91 ± 0.02	1.89 ± 0.02	2.98 ± 0.03	6.37 ± 0.20
*ALAD1-2*/*2-2*	8.8	3.14 ± 0.30	0.90 ± 0.04	1.96 ± 0.04	3.11 ± 0.12	6.32 ± 0.63

* *p* < 0.05.

**Table 4:** Adjusted POR^a^ (95% CI) for hypertension stratified by race/ethnicity.

Variable	Non-Hispanic whites^b^	Non-Hispanic blacks^b^	Mexican Americans^b^
BLL quartile			
0.7–1.4 μg/dL	Reference	Reference	Reference
1.5–2.3 μg/dL	1.21 (0.66–2.24)	1.83 (1.08–3.09)	0.74 (0.24–2.23)
2.4–3.7 μg/dL	1.57 (0.88–2.80)	2.38 (1.40–4.06)	1.43 (0.61–3.38)
3.8–52.9 μg/dL	1.52 (0.80–2.88)	2.92 (1.58–5.41)	1.27 (0.59–2.75)
*ALAD1-2*/*2-2*	0.76 (0.17–3.50)	3.40 (0.05–219.03)	0.49 (0.08–3.20)
*ALAD1-1*	Reference	Reference	Reference

a Adjusted for race/ethnicity, age, sex, BMI, alcohol ingestion, smoking status, education, serum creatinine, serum total calcium, glycosylated hemoglobin, and hematocrit.

b Adjusted for age, sex, BMI, alcohol ingestion, smoking status, education, serum creatinine, serum total calcium, glycosylated hemoglobin, and hematocrit.

**Table 5:** Adjusted POR^a^ (95% CI) for hypertension by *ALAD2* allele within BLL quartiles in the NHANES III DNA bank stratified by race/ethnicity.

Characteristic	Quartile 1 [0.7–1.4 μg/dL (*n* = 1,206)]	Quartile 2 [1.5–2.3 μg/dL (*n* = 1,263)]	Quartile 3 [2.4–3.7 μg/dL (*n* = 1,388)]	Quartile 4 [3.8–52.9 μg/dL (*n* = 1,657)]
Non-Hispanic whites^b^	0.78 (0.15–4.08)	1.11 (0.45–2.69)	0.67 (0.39–1.12)	2.00 (1.12–3.55)
Non-Hispanic blacks^b^	5.71 (0.04–745.01)	3.13 (0.48–20.50)	1.31 (0.29–5.88)	0.60 (0.22–1.63)
Mexican Americans^b^	0.34 (0.05–2.11)	1.62 (0.43–6.13)	0.94 (0.24–3.73)	0.92 (0.30–2.86)

a Adjusted for race/ethnicity, age, sex, BMI, alcohol ingestion, smoking status, education, serum creatinine, serum total calcium, glycosylated hemoglobin, and hematocrit.

b Adjusted for age, sex, BMI, alcohol ingestion, smoking status, education, serum creatinine, serum total calcium, glycosylated hemoglobin, and hematocrit.

**Table 6:** Multivariate linear regression models for systolic and diastolic BP for individuals who were not currently taking medications for high BP stratified by race/ethnicity (β-coefficient ± SE).^a^

	Non-Hispanic whites		Non-Hispanic blacks		Mexican Americans	
Characteristic	Full model	*p*-Value	Full model	*p*-Value	Full model	*p*-Value
Systolic BP						
Ln BLL	1.05 ± 0.37	0.01	2.55 ± 0.49	0.001	0.84 ± 0.46	0.08
*ALAD1-2*/*2-2*	−2.14 ± 1.19	0.09	6.85 ± 2.92	0.03	0.84 ± 0.95	0.38
Ln BLL * *ALAD1-2*/*2-2*^b^	2.51 ± 1.16	0.04	−8.41 ± 3.31	0.02	−0.86 ± 1.03	0.41
Diastolic BP						
Ln BLL	−0.14 ± 0.49	0.77	1.99 ± 0.44	0.0002	0.74 ± 0.38	0.06
*ALAD1-2*/*2-2*	−1.18 ± 0.92	0.21	5.36 ± 4.41	0.24	0.89 ± 1.08	0.42
Ln BLL * *ALAD1-2*/*2-2*^b^	0.74 ± 1.08	0.50	−7.20 ± 3.61	0.06	−2.22 ± 1.08	0.052

a Adjusted for age, sex, BMI, alcohol ingestion, smoking status, education, serum creatinine, serum total calcium, glycosylated hemoglobin, and hematocrit.

b The interaction term of Ln BLL and ALAD genotype.
