# Errata

**Published:** 2010-10

Scinicariello et al. have reported two text errors in their article “Modification by ALAD of the Association between Blood Lead and Blood Pressure in the U.S. Population: Results from the Third National Health and Nutrition Examination Survey” [ Environ Health Perspect 118:259–264 (2010)].

First, in the third paragraph of their article (p. 259), the sentence summarizing results of a study of Korean lead smelter workers should have been as follows:

A study conducted among Korean lead smelter workers (*n* = 798; mean BLL = 32.0 μg/dL) found that the *ALAD* polymorphism did not change the association between blood lead and hypertension at occupational exposure levels compared with *ALAD1* homozygous carriers (Lee et al. 2001).

Second, the sentence in the third paragraph of the “Discussion” (p. 262) was incorrect. The corrected sentence is as follows:

Two previous studies on *ALAD*, BLL, and BP—one conducted among occupationally exposed workers (Lee et al. 2001) and the other conducted at lower lead exposure level (Smith et al. 1995)—found no association of *ALAD* polymorphism and BP outcomes.

The authors apologize for the errors.

